# Variation in readmission and mortality following hospitalisation with a diagnosis of heart failure: prospective cohort study using linked data

**DOI:** 10.1186/s12913-017-2152-0

**Published:** 2017-03-21

**Authors:** Rosemary J. Korda, Wei Du, Cathy Day, Karen Page, Peter S. Macdonald, Emily Banks

**Affiliations:** 1 National Centre for Epidemiology and Population Health, Research School of Population Health, Australian National University, Canberra, Australia; 2 Deakin University, School of Nursing and Midwifery, Melbourne, Australia; 3 St Vincent’s Clinical School, Faculty of Medicine, University of New South Wales, Kensington, Australia; 4 The Sax Institute, Sydney, Australia

**Keywords:** Heart failure, Readmissions, Mortality, Health services, Health systems, Multilevel model, Linked data

## Abstract

**Background:**

Hospitalisation for heart failure is common and post-discharge outcomes, including readmission and mortality, are often poor and are poorly understood. The purpose of this study was to examine patient- and hospital-level variation in the risk of 30-day unplanned readmission and mortality following discharge from hospital with a diagnosis of heart failure.

**Methods:**

Prospective cohort study using data from the Sax Institute’s 45 and Up Study, linking baseline survey (Jan 2006-April 2009) to hospital and mortality data (to Dec 2011). Primary outcomes in those admitted to hospital with heart failure included unplanned readmission, mortality and combined unplanned readmission/mortality, within 30 days of discharge. Multilevel models quantified the variation in outcomes between hospitals and examined associations with patient- and hospital-level characteristics.

**Results:**

There were 5074 participants with a heart failure admission discharged from 251 hospitals; 1052 (21%) had unplanned readmissions, 186 (3.7%) died, and 1146 (23%) had either/both outcomes within 30 days of discharge. Crude outcomes varied across hospitals, but between-hospital variation explained little of the total variation in outcomes (intraclass correlation coefficients (ICC) after inclusion of patient factors: 30-day unplanned readmission ICC = 0.0125 (*p* = 0.24); death ICC = 0.0000 (*p* > 0.99); unplanned readmission/death ICC = 0.0266 (*p* = 0.07)). Patient characteristics associated with a higher risk of unplanned readmission included: being male (male vs female, adjusted odds ratio (aOR) = 1.18, 95% CI: 1.00–1.37); prior hospitalisation for cardiovascular disease (aOR = 1.44, 1.08–1.91) and for anemia (aOR = 1.36, 1.14–1.63); comorbidities at admission (severe vs none: aOR = 1.26, 1.03–1.54); lower body-mass-index (obese vs normal weight: aOR = 0.77, 0.63–0.94); and lower social interaction scores. Similarly, risk of 30-day mortality was associated with patient- rather than hospital-level factors, in particular age (≥85y vs 45–< 75y: aOR = 3.23, 1.93–5.41) and comorbidity (severe vs none: aOR = 2.68, 1.82–3.94).

**Conclusions:**

The issue of high readmission and mortality rates in people with heart failure appear to be system-wide, with the variation in these outcomes essentially attributable to variation between patients rather than hospitals. The findings suggest that there are limitations in using these outcomes as hospital performance measures in this patient population and support the need for patient-centred strategies to optimise heart failure management and outcomes.

**Electronic supplementary material:**

The online version of this article (doi:10.1186/s12913-017-2152-0) contains supplementary material, which is available to authorized users.

## Background

Heart failure is a major health problem in high income countries; in Australia an estimated 2.8% of the total population aged 45 years and older are affected [1]. Although treatment for heart failure has improved and both mortality and hospital rates have been declining [2–5], the annual rate of hospitalisation for this condition remains relatively high; it was the primary reason for admission to hospital in over 47 thousand admissions in Australia in 2013–14 [6]. Of particular concern is the high hospital readmission rate, with around one in every four or five patients admitted to hospital with a heart failure diagnosis being readmitted within one-month of discharge—three-quarters within one year [5, 7]. Mortality rates following admission to hospital for heart failure are also high, although possibly declining over time [4], with around one in ten dying within one month of admission for heart failure and one quarter within a year [4, 5]. Similar heart failure prevalences and high readmission and mortality rates are observed in other countries [8–13].

Rates of death and readmission—particularly unplanned returns to hospital—within one month of hospital discharge are used as hospital performance measures, both nationally and internationally [6, 14–16]. These measures can reflect the quality of care provided in hospital and access to appropriate follow-up after discharge, thus providing an indication that patient care could be improved and/or that more efficient use could be made of available resources [14]. However, it is recognised that not all readmissions and deaths are avoidable, with the risks also relating to individual patient characteristics such as age and comorbidity. Moreover, to understand hospital variation in heart failure outcomes requires a quantitative understanding of the contributions of variation at both the hospital and patient level. A recent Australian report has investigated the distribution of these performance measures according to hospital, risk adjusting for patient age, sex and comorbidities, with an emphasis on those hospitals which are “outliers” [14]; and other Australian studies have examined patient-level risk factors derived from linked hospital records [5, 7, 11, 17, 18]. However, there remains a lack of large-scale quantitative data that quantify both patient and hospital-level variation in post-discharge heart failure outcomes, and associations with patient characteristics.

The purpose of this study was to use linked population-based survey, hospital and death data to examine patient- and hospital-level variation in the risk of 30-day unplanned readmission and 30-day mortality among those aged 45 years and over discharged from hospital with a diagnosis of heart failure. Specifically, using multilevel modelling, the study aimed to: quantify the variation in outcomes—unplanned readmissions and mortality—across hospitals; examine the extent to which selected patient, heart failure admission and hospital characteristics explain this variation; and quantify the strength of association between the outcomes and patient and hospital characteristics.

## Methods

### Data sources and study population

We used data from the Sax Institute’s 45 and Up Study, a prospective cohort study involving 267,153 men and women aged 45 years and over from New South Wales (NSW). Participants in the Study were randomly sampled from the database of Medicare Australia, with over-sampling, by a factor of two, of individuals aged 80 years and over and residents in rural areas. Around 10% of the entire NSW population aged 45 years and over was included in the cohort [19]. Participants joined the study by completing a baseline questionnaire, distributed between 1 January 2006 and 31 December 2008, and provided signed consent for linkage of their information to a range of health-related databases. The 45 and Up Study is described in detail elsewhere [19], and questionnaires can be viewed at https://www.saxinstitute.org.au/our-work/45-up-study/questionnaires.

Questionnaire data from study participants have been linked probabilistically to the NSW Admitted Patient Data Collection (APDC) and the NSW Register of Births, Deaths and Marriages (RBDM) by the Centre for Health Record Linkage. The APDC is a census of all public and private hospital admissions in NSW. The linked data for this study contain details of admissions for participants from 1 January 2000 to 31 December 2011, including dates of admission and discharge, the primary reason for admission and up to 54 additional clinical diagnoses coded using the International Classification of Diseases 10th revision (ICD-10) Australian Modification [20], whether the admission was planned or unplanned, discharge status and hospital type. Linked death data were also available until December 2011. Death registrations capture all deaths in NSW; date, but not cause, of death information was available for this study.

### Study sample

**Fig. 1:**
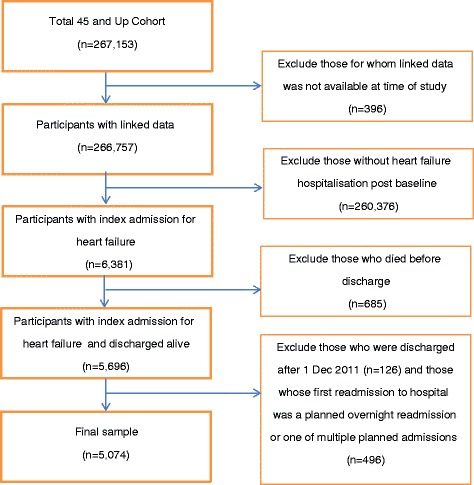
Study population flow chart of sample exclusions

The study sample comprised 45 and Up Study participants with linked data and who had at least one admission with a heart failure diagnosis recorded following entry into the Study (ICD-10 codes: I50, I11.0, I13.0, I13.2). We included those with heart failure recorded as either a primary diagnosis or as an additional diagnosis in any of the 54 additional diagnosis fields. The first admission following entry into the study in which a heart failure diagnosis was recorded is hereafter referred to as the index admission. We excluded those who died before being discharged from hospital and those who did not have at least 30 days of follow-up time (i.e. patients who were discharged from the index admission after 1 Dec 2011). We also excluded those whose first readmission to hospital within the 30-day follow-up was a planned overnight readmission or was one of multiple planned admissions (overnight or same day); these exclusion criteria were applied as these planned admissions are likely to affect the risk of an unplanned admission. See Fig. 1 for a flow chart of sample selection.

Participants were followed for 30 days from the date of discharge from their index admission. Over the relatively short follow-up period, a small but unknown number of participants are likely to have moved out of NSW; among those continuing to reside in NSW, follow-up for hospitalisations is considered to be ~98% complete [21]. Quality assurance data on the data linkage show false positive and negative rates of <0.5 and <0.1%, respectively.

### Outcomes: readmissions and deaths

The main outcomes were (a) *30-day unplanned readmission*: unplanned readmission (emergency admission type) within 30 days of discharge of the index admission for any cause; and (b) *30-day mortality*: death from any cause within 30 days of discharge of the index admission; and (c) due to the likelihood of competing risks, we also included a combined readmission or mortality outcome, *30-day unplanned readmission/mortality*.

The linked APDC records were used to identify the index admission, discharge status (dead or alive), discharge hospital type and unplanned readmissions. The linked RBDM records were used to identify deaths post discharge. The date of discharge was taken as time zero for time-to-event calculations. Where an episode of care ended with transfers (determined from admission and separation dates of consecutive admissions), we treated all consecutive episodes as a nested care, and thus, the date of discharge was the date of discharge from the last hospital in which the nested care ended; similarly, hospital characteristics were based on the hospital from which the patient was discharged.

### Exposures: patient, index admission and hospital characteristics

Sociodemographic information and most of the baseline health information was self-reported on the baseline questionnaire (apart from area of residence, which was derived from postcodes obtained from Medicare data). Other information on health status, including previous hospital admission diagnoses, and information on index admission and hospital characteristics, was obtained from the APDC records.

Sociodemographic variables, self-reported on the baseline questionnaire, included: age, calculated as age at admission (categorised as 45– < 75, 75– < 85, ≥85 years); sex (male, female); area of residence (major cities, inner regional, more remote, based on the Accessibility/ Remoteness Index of Australia Plus [22] score associated with the postcode of residence); marital status (single, defacto/married); language other than English spoken at home (yes, no); education (highest qualification categorised as no school certificate, school/trade certificate or diploma, tertiary degree); income (pre-tax annual household annual household income from all sources including benefits, pensions and superannuation, categorised as < $20,000, $20,000–39,999, $40,000–69,999, ≥$70,000) and private health insurance (yes, no).

Baseline health variables self-reported on the questionnaire included: smoking (never, past, current); alcohol intake (0, 1– < 15, ≥15 drinks per week); body mass index (BMI) (underweight (<18 kg/m^2^), normal weight (18– < 25 kg/m^2^), overweight (25– < 30 kg/m^2^), obese (30– < 50 kg/m^2^)); and excellent memory (yes, no). Heart disease (yes, no) and diabetes (yes, no) was determined by asking the participant if a doctor had ever told them that they had that condition. Physical functioning was assessed using the Medical Outcomes Study-Physical Functioning [23]. The scale assesses functional scale capacity by inquiring about an individual’s ability to perform a range of moderate and vigorous physical tasks as well as everyday activities. The total PF-10 score ranges from 0 to 100 and was categorised as severe physical functional limitation (<75) or not (≥75). Psychological distress was determined using the Kessler Psychological Distress Scale (K10) [24]. All items on the K10 begin with the phrase ‘during the past 4 weeks, about how often did you feel (…)’ followed by the description of an emotional state, such as: ‘tired out for no good reason?’ All answer options were based on a 5-point scale (‘none of the time’ (1), through to, ‘all of the time’ (5)). The K10 score has a range from 10 to 50 and was categorised as low (<16), medium (16– < 22), high (22–30) and very high (≥30). We also calculated a social interaction score, based on items similar to those used in the social interaction subscale score of the Duke Social Support Index [25, 26]. This subscale has four items, with the total sub-scale score ranging from 4 to 12, with higher scores indicating more social interaction.

Baseline health variables derived from APDC records included prior hospitalisation in the 6 years prior to baseline for each of the following conditions: heart failure, other major cardiovascular disease (I11-I13 [excl. I11.0, I13.0 and I13.2]; I20-I28, I34-36, I42, I44-I49, I51, I61-I67, I69, I70-I77, I80, G45, G46) [27], renal disease (I12.0, I13.1, N03.2 N03.7, N05.2-N05.7, N18, N19, N25.0, Z49.0, Z94.0, Z99.2), anaemia (D50-D53, D55-59-D66), atrial fibrillation (I48) and dementia (F00-F03, G30, G31.0, G31.1, G31.8), as identified in any of the 55 diagnosis fields. The time frame of pre-enrolment hospitalisation history was set to 6 years as this was the maximum retrospective follow-up available for the whole study population.

Index admission characteristics, derived from APDC records, included heart failure primary diagnosis, classified as either yes, i.e. diagnosis of heart failure recorded in the primary diagnosis field, or no, i.e. heart failure diagnosis recorded only in one of the 54 secondary diagnosis fields; whether unplanned admission or not (based on urgency of admission status); comorbidity using the Charlson index (i.e., sum of death propensity scores assigned to 16 conditions including pulmonary disease, diabetes, myocardial infarction, peripheral vascular disease, cerebral vascular disease, cancer, and liver disease) [28] for the index admission (categorised as none: total score of 0; mild-moderate: total score of 1 or 2; or severe: total score ≥2); length of stay, calculated as separation date minus admission date, plus one day for same day admissions (categorised as 1, 2–6, ≥7); prior hospitalisation days, calculated as the number of days spent in hospital in the 12 months prior to the index admission; and prior hospitalisation-admissions, calculated as the number of admissions in the 12 months prior to the index admission.

Hospital characteristics included hospital type, based on the Australian Institute of Health and Welfare peer group classification system, which categorises hospitals captured in the APDC using type and nature of the services provided in addition to casemix-adjusted separations and geographic locations [29]; we further categorised peer groups as principal referral (classification A), major city (classification B), medium district (classification C), small community (classification D), private hospital group, or other (classifications E, F, G). We also considered characterising hospitals in relation to remoteness and hospital size; however these characteristics are essentially captured in the peer group classification. In addition, we classified hospitals according to whether they had a dedicated heart failure service, using information compiled in the *Heart Foundation’s Directory of NSW/ACT Cardiovascular Health Services (Version 15, Jan 2014)*.

### Statistical analysis

For descriptive data, numbers and proportions (risks) are presented, where appropriate. We graphed crude risks of readmission and death (with 95% confidence intervals) in increasing order by hospital, excluding hospitals with fewer than 5 index admissions. To allow for the small number of index admissions in some hospitals, we used the Agresti-Coull correction [30]. We also used Kaplan-Meier curves to present the elapsed time from date of discharge until the occurrence of death or first readmission graphically by hospital groups and nonparametric log-rank tests to detect differences between hospital groups.

For the main analyses, we used multilevel logistic regression models, with patients (first level) nested in hospitals (second level). These models account for possible patient clustering within hospitals and enable hospital variation in the risk of readmission or death to be quantified (random effects), as well as estimation of the strength of the associations between these outcomes and patient and hospital characteristics (fixed effects), after accounting for hospital-level variation.

To examine the associations between the outcomes and all of the patient, admission and hospital characteristics, we first ran age and sex adjusted analyses. These analyses were used to guide which characteristics to include in the final multilevel models. We included the following variables in the full-risk-adjusted model: age, sex, smoking status, and previous hospitalisation for heart failure, other major cardiovascular disease, dementia, anaemia and renal disease; and of the other individual covariates, we retained those with a p-value less than 0.25 in the age and sex adjusted analyses and/or causing a change in the effect size of 10% or more. To reduce the impact of collinearity, we omitted length of stay and used the Charlson Index as the single proxy of index case severity. We used Type III tests of fixed effects (between and within method) for modelled predictors.

We ran a series of multilevel models for each of the outcomes, using five model specifications. The first was a null model (i.e. it did not include any patient or hospital-level exposure variables), with only random intercepts to examine if there was significant variation in readmission or mortality across hospitals (Model 1). In subsequent models we sequentially added covariates to the previous model: Model 2, age and sex; Model 3, additional patient sociodemographic variables; Model 4, patient health and index admission characteristics; and in Model 5, hospital level variables. Missing values for categorical covariates were included in the models as separate categories, except for the continuous Duke Social Support Index, where missing values were assigned the median value (with this having little impact on the regression coefficient point estimate while maintaining the sample size as a conservative substitute for imputation).

Hospital-level variation in outcomes was estimated in terms of variance, and a range of statistical terms were used to report between-hospital variation (random effects), including: the intraclass correlation coefficient (ICC), used an estimate of the proportion of overall variation in outcomes explained by variation between hospitals; the proportional change in variance, which is the percentage change in the variance with the addition of variables into the model; and the median odds ratio, which quantifies hospital variance in terms of odds ratios, describing the median increased individual risk associated with moving to a hospital with higher risk [31, 32]. We estimated the associations between patient, admission and hospital characteristics and study outcomes (fixed effects) in terms of odds ratios (ORs), with 95% confidence intervals (CI). P-values less than 0.05 were considered statistically significant.

We also ran a series of sensitivity analyses. We examined the effects of excluding index hospitalisations for patients with previous hospitalisations for heart failure, and of including only index hospitalisations where heart failure was the primary diagnosis. We considered restricting readmissions to heart failure admissions only; however there was an insufficient number of cases to proceed with these analyses.

The study adhered to STROBE guidelines. We used SAS 9.4 package (SAS Institute, Cary, NC) to perform all data analyses.

## Results

### Sample description

At the time of this study, linkages could be made for 266,757/267,153 (99% of participants) in the 45 and Up Study cohort. Among these participants, a total of 6381 index admissions for heart failure were identified (>99% with ICD-10 code I50). Compared to the other cohort members, those with a heart failure admission were older (9% vs 45% aged ≥85 years, respectively), were more likely to be male (48% vs 58%) and had poorer health at baseline (self-reported poor health: 2.6% vs 12%).

After excluding 685 patients (11%) who died before discharge and other exclusions (see Fig. 1), there were 5074 patients/index admissions in the final sample. Of these, 1973 (39%) were for a primary diagnosis of heart failure; 4180 admissions (82%) of total index admissions were unplanned. The majority of patients (58%) were male, and age at index admission ranged from 48 to >100 years, median 82 years (IQR: 12). In terms of baseline health, almost a quarter (23%) had at least one hospital admission with a diagnosis of heart failure recorded in the 6 years prior to baseline, and a large proportion (92%) had at least one prior admission for another major cardiovascular disease. Of those with known physical functioning scores, more than two-thirds (70%) were classified as having severe limitations at baseline. At the time of the index admission, 28% of patients had Charlson Index scores indicating moderate comorbidity and 15% severe comorbidity. Patients were discharged from 251 different hospitals, with half (49%) of all discharges from principal referral hospitals. Further sample characteristics are shown in Table 1 and additional hospital characteristics are in Additional file 1: Table S1.

**Table 1:** Sample characteristics and proportions^a^ who were readmitted or died

Variables	Total sample		30-day unplanned readmission (%)^a^	30-day mortality (%)^a^
	n	%^a^		
Sociodemographic				
Age (years, at index admission)				
45– < 75	1303	26	20	1.8
75– < 85	2142	42	22	3.5
≥ 85	1629	32	20	5.5
*Mean (SD)*		80 (9.4)	80 (8.8)	84 (8.4)
Sex				
Male	2927	58	22	4.0
Female	2147	42	19	3.2
Language other than English				
Yes	559	11	21	3.4
No	4515	89	21	3.7
Marital status				
Married	2864	56	20	3.6
Not married	2210	44	21	3.8
Region of residence				
Major cities	2535	50	20	4.1
Regional	1566	31	21	3.5
Remote	973	19	22	2.9
Education				
No school certificate	1134	23	21	3.8
School/Trade certificate, diploma	3211	66	22	3.5
Tertiary degree	524	11	18	5.0
Unknown	205			
Household income (AUD)				
< 20,000	1994	57	31	5.5
20,000–39,000	914	26	28	5.7
40,000–69,999	363	10	28	4.8
≥ 70,000	220	6.3	26	4.0
Unknown	1583			
Private health insurance				
Yes	2131	42	19	3.8
No	2943	58	22	3.6
Baseline health (self-reported)				
Previous heart disease				
Yes	2408	47	21	3.5
No	2666	53	20	3.8
Previous diabetes				
Yes	1213	24	21	3.1
No	3861	76	21	3.9
Excellent memory				
Yes	505	10	21	3.0
No	4569	90	21	3.7
Physical functioning score				
0– < 75 (severe)	2662	70	28	5.8
≥ 75	1127	30	25	2.7
Unknown	1285			
Duke Social Support Index				
*Mean (SD)*		8.7(1.6)	8.6 (1.6)	8.6 (1.7)
Psychological distress				
Very low	3478	74	22	4.5
Low	770	16	24	2.2
Medium	312	6.6	27	4.5
High	140	3	25	3.1
Unknown	374			
BMI				
Underweight	128	2.8	27	7.9
Normal	1577	35	24	4.8
Overweight	1616	36	24	3.8
Obese	1191	26	20	2.3
Unknown	562			
Smoking status				
Current smoker	273	5.4	26	5.2
Ex-smoker	2384	47	21	3.4
Never smoker	2388	47	20	3.8
Unknown	29			
Alcohol (drinks per week)				
None	2230	46	22	3.8
1–14 drinks	2083	43	22	4.3
≥ 15 drinks	522	11	20	2.8
Unknown	239			
Hospitalisation history (Admitted Patient Data Collection, 6 year prior to baseline)				
Previous CVD				
Yes	4669	92	21	3.6
No	405	8	15	4.4
Previous heart failure				
Yes	1176	23	24	4.2
No	3898	77	20	3.5
Previous renal disease				
Yes	564	11	26	3.9
No	4510	89	20	3.6
Previous anaemia				
Yes	900	18	26	4.6
No	4174	82	20	3.5
Previous atrial fibrillation				
Yes	1422	28	22	3.2
No	3652	72	20	3.8
Previous dementia				
Yes	73	1.4	18	6.9
No	5001	99	21	3.6
Hospitalisation history (Admitted Patient Data Collection, 12 months prior to index admission)				
No. of admissions (last 12 months)				
*Mean (SD)*		1.3 (2.9)	1.9 (5.0)	1.8 (3.4)
No. of hospital days (last 12 months)				
*Mean (SD)*		7.0 (15)	9.2 (18)	11 (18)
Index admission characteristics				
Primary diagnosis				
Yes	1973	39	20	2.3
No	3101	61	21	4.6
Unplanned admission				
Yes	4180	82	22	3.8
No	894	18	16	3.2
Length of stay (days)				
1	450	8.9	18	2.7
2–6	1922	38	20	2.5
≥ 7	2702	53	21	4.7
Current comorbidity (Charlson Index)				
Minor	2933	58	19	2.7
Moderate	1403	28	22	4.0
Severe	738	15	25	6.8
Length of stay (days)				
Median (IQR)		7 (10)	7 (12)	12 (18)
Hospital characteristics				
Hospital peer group				
Principal referral	2493	49	22	3.4
Major city	537	11	20	5.0
District	606	12	21	4.1
Community	355	7	26	3.4
Private	900	18	17	3.7
Others	183	3.6	17	2.7
Heart failure service				
Yes	1695	33	22	3.6
No	3379	67	20	3.7
Totals	5074	100	21	3.7

^a^Proportions (%), or means and standard deviations [SD] or median and interquartile range (IQR) for continuous variables. Denominators for percentages are the total known (i.e. non-missing/valid) values

### Risk of 30-day readmission and death

Overall, 21% (1052/5074) of the patients with heart failure discharged from hospital had unplanned readmissions, 3.7% (*n* = 186) died within 30 days of discharge and 23% (*n* = 1146) had either or both outcomes (readmission/death).

#### Between-hospital variation in risk of readmission and/or death

**Fig. 2:**
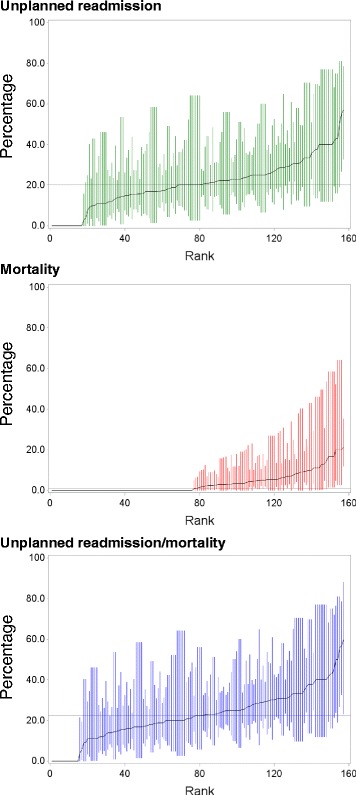
Crude risks (%, with 95% confidence intervals) of 30-day readmission and mortality by individual hospital, ranked from lowest to highest, for the 157 hospitals with at least five index admissions

A number of hospitals had no readmissions (74/251, 30%) or deaths (166/251, 66%) within 30 days of discharge in the period of interest. The crude risks, reported for the 157 hospitals (63%) that had at least 5 index admissions (accounting for 96% of the total admissions), ranged from 0 to 57% (median = 20; IQR = 11) for 30-day unplanned readmission; from 0 to 21% (median = 1.1; IQR = 5.3) for 30-day mortality and from 0 to 60% (median = 22; IQR = 14) for 30-day readmission/mortality (see Fig. 2).

**Fig. 3:**
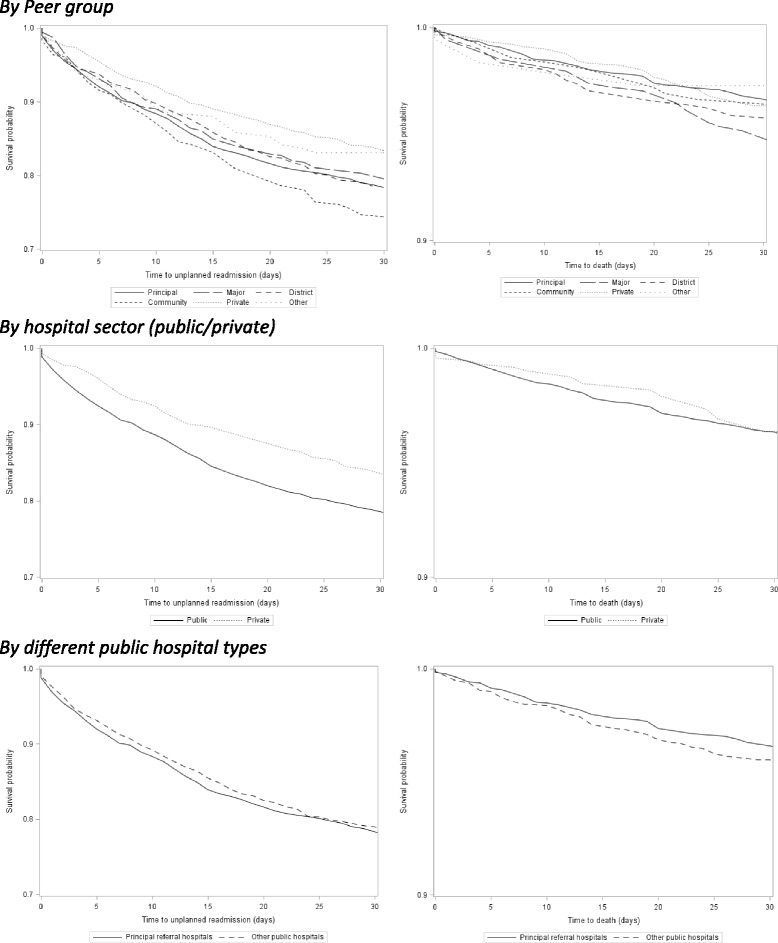
Time to unplanned readmission and death, by hospital type

Time to unplanned readmission and to death according to discharge hospital type is shown in Fig. 3. The probability of unplanned readmission (log-rank test, *p* < 0.001), but not survival (*p* = 0.061), differed significantly across hospital peer groups. Patients discharged from public hospitals had a higher probability of unplanned readmission, compared with those admitted to private hospitals (*p* < 0.001); among public hospitals, there was no significant difference in the probability of unplanned readmissions between the principal referral hospitals and other hospital types (*p* = 0.48).

**Fig. 4:**
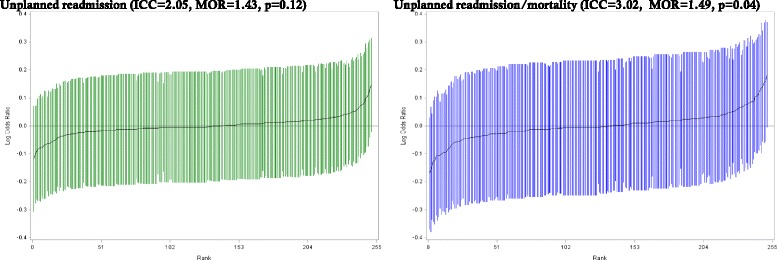
Odds of 30-day unplanned readmission and unplanned readmission/mortality (logarithmic scale) for each hospital, ranked from lowest to highest, with 95% confidence intervals

Multilevel modelling (Fig. 4; and Table 2–30-day unplanned readmission; Table 3–30-day mortality; and Additional file 2: Table S2–30-day unplanned readmission/mortality) showed the proportion of overall variation in outcomes explained by variation between hospitals (Model 1) was not statistically significant for unplanned readmission (ICC = .0205, *p* = 0.12) or mortality (ICC = 0.0000), while the between-hospital variation in the combined readmission/mortality outcome was marginally significant in the null model, explaining 3.0% of the total variance in outcomes (*p* = 0.04). After adjusting for age and sex (Model 2), there was little change in the between-hospital hospital variation in any of the outcomes. There was a gradual reduction in the proportion of variance explained at the hospital level in unplanned readmissions and unplanned readmission/mortality after adjusting for additional sociodemographic variables (Model 3) and patient health and admission characteristics (Model 4), with between-hospital variation not statistically significant in these models.

**Table 2:** Multilevel model results for 30-day unplanned readmission: random effect measures and adjusted odds ratios (95%CIs) for individual-level and hospital-level variables

Model	1	2	3	4	5
Random effects					
Hospital variance	0.14	0.15	0.13	0.11	0.09
ICC	0.0205	0.0212	0.0172	0.0125	0.0074
P-value	0.12	0.12	0.17	0.24	0.34
MOR	1.43	1.44	1.41	1.37	1.32
PCV (%)		−1.82	9.95	14.94	22.9
Fixed effects	Odds ratios				
Sociodemographic					
Age (years, at index admission)					
45– < 75		1.00	1.00	1.00	1.00
75– < 85		1.10 (0.93,1.31)	1.10 (0.93,1.31)	1.06 (0.89,1.27)	1.06 (0.89,1.27)
≥ 85		1.01 (0.84,1.21)	0.99 (0.82,1.19)	0.92 (0.75,1.13)	0.92 (0.75,1.13)
Sex					
Male		1.19 (1.04,1.37)	1.23 (1.07,1.43)	1.17 (1.00,1.37)	1.18 (1.00,1.37)
Female		1.00	1.00	1.00	1.00
Marital status					
Married			0.90 (0.77,1.04)	0.90 (0.78,1.05)	0.91 (0.78,1.05)
Not married			1.00	1.00	1.00
Region of residence					
Major cities			1.00	1.00	1.00
Regional			1.01 (0.86,1.19)	1.03 (0.87,1.22)	1.03 (0.87,1.22)
Remote			1.08 (0.90,1.31)	1.13 (0.93,1.36)	1.04 (0.83,1.29)
Education					
No school certificate			1.00	1.00	1.00
School/trade/certificate/diploma			1.11 (0.94,1.32)	1.13 (0.95,1.35)	1.14 (0.96,1.36)
Tertiary degree			0.89 (0.67,1.17)	0.89 (0.67,1.18)	0.91 (0.69,1.21)
Private health insurance					
Yes			0.89 (0.77,1.03)	0.92 (0.80,1.07)	0.97 (0.83,1.14)
No			1.00	1.00	1.00
Baseline health (self-reported)					
Duke Social Support Index				0.95 (0.91,0.99)	0.95 (0.91,0.99)
Body mass index					
Underweight (15– < 18 kg/m^2^)				1.23 (0.80,1.89)	1.23 (0.80,1.89)
Normal (18– < 25 kg/m^2^)				1.00	1.00
Overweight (25– < 30 kg/m^2^)				0.98 (0.83,1.17)	0.98 (0.82,1.17)
Obese (>30–50 kg/m^2^)				0.78 (0.64,0.95)	0.77 (0.63,0.94)
Smoking status					
Current smoker				1.00	1.00
Ex-smoker				0.76 (0.57,1.03)	0.78 (0.57,1.05)
Never smoker				0.76 (0.56,1.03)	0.77 (0.57,1.05)
Hospitalisation history (APDC, prior 6 years)					
Previous CVD					
Yes				1.41 (1.06,1.88)	1.44 (1.08,1.91)
No				1.00	1.00
Previous heart failure					
Yes				1.12 (0.94,1.32)	1.11 (0.93,1.31)
No				1.00	1.00
Previous renal disease					
Yes				1.12 (0.89,1.40)	1.11 (0.89,1.39)
No				1.00	1.00
Previous anaemia					
Yes				1.36 (1.13,1.62)	1.36 (1.14,1.63)
No				1.00	1.00
Previous dementia					
Yes				0.66 (0.35,1.22)	0.66 (0.36,1.22)
No				1.00	1.00
Index admission characteristics					
Primary diagnosis					
Yes				0.94 (0.81,1.08)	0.93 (0.80,1.07)
No				1.00	1.00
Current comorbidity (Charlson Index)					
None				1.00	1.00
Mild-moderate				1.15 (0.98,1.35)	1.15 (0.98,1.35)
Severe				1.25 (1.02,1.52)	1.26 (1.03,1.54)
Hospital characteristics					
Hospital peer group					
Principal referral					1.00
Major city					0.92 (0.72,1.18)
District					1.03 (0.81,1.31)
Community					1.34 (0.99,1.81)
Private					0.80 (0.63,1.01)
Others					0.72 (0.48,1.09)
Heart failure service					
Yes					1.10 (0.93,1.31)
No					1.00

*ICC* intraclass correlation coefficient, *MOR* median odds ratio, *PCV* proportional change in variance

**Table 3:** Multilevel model results for 30-day mortality: random effect measures and adjusted odds ratios (95% CIs) for individual-level and hospital-level variables

Model	1	2	3	4	5
Random effects					
Hospital variance	0	0	0	0	0
ICC	0	0	0	0	0
Fixed effects	Odds ratios				
Sociodemographic					
Age (years, at index admission)					
45– < 75		1.00	1.00	1.00	1.00
75– < 85		1.99 (1.24,3.20)	1.97 (1.22,3.16)	2.07 (1.26,3.40)	2.04 (1.24,3.35)
≥ 85		3.33 (2.09,5.30)	3.23 (2.00,5.20)	3.30 (1.97,5.52)	3.23 (1.93,5.41)
Sex					
Male		1.36 (1.00,1.84)	1.34 (0.96,1.85)	1.36 (0.96,1.93)	1.36 (0.96,1.93)
Female		1.00	1.00	1.00	1.00
Marital status					
Married			1.02 (0.74,1.40)	1.06 (0.77,1.47)	1.06 (0.77,1.47)
Not married			1.00	1.00	1.00
Region of residence					
Major cities			1.00	1.00	1.00
Regional			1.01 (0.72,1.41)	1.06 (0.75,1.50)	1.03 (0.72,1.46)
Remote			0.85 (0.55,1.31)	0.90 (0.58,1.40)	0.83 (0.51,1.38)
Education					
No school certificate			1.00	1.00	1.00
School/trade/certificate/diploma			0.90 (0.62,1.31)	0.91 (0.62,1.32)	0.91 (0.62,1.32)
Tertiary degree			1.18 (0.69,2.01)	1.15 (0.67,1.98)	1.16 (0.67,1.99)
Private health insurance					
Yes			1.05 (0.77,1.43)	1.10 (0.80,1.51)	1.12 (0.80,1.56)
No			1.00	1.00	1.00
Baseline health (self-reported)					
Duke Social Support Index				0.97 (0.88,1.07)	0.97 (0.88,1.07)
BMI					
Underweight				1.68 (0.80,3.52)	1.63 (0.77,3.42)
Normal				1.00	1.00
Overweight				0.90 (0.62,1.30)	0.90 (0.62,1.31)
Obese				0.62 (0.38,1.02)	0.61 (0.37,1.01)
Smoking status					
Current smoker				1.00	1.00
Ex-smoker				0.50 (0.27,0.94)	0.49 (0.27,0.92)
Never smoker				0.58 (0.31,1.09)	0.57 (0.31,1.07)
Hospitalisation history (APDC, prior 6 years)					
Previous CVD					
Yes				0.73 (0.43,1.22)	0.74 (0.44,1.24)
No				1.00	1.00
Previous heart failure					
Yes				1.23 (0.85,1.77)	1.22 (0.84,1.76)
No				1.00	1.00
Previous renal disease					
Yes				0.73 (0.44,1.21)	0.73 (0.44,1.21)
No				1.00	1.00
Previous anaemia					
Yes				1.17 (0.80,1.72)	1.18 (0.81,1.72)
No				1.00	1.00
Previous dementia					
Yes				1.31 (0.50,3.40)	1.30 (0.50,3.36)
No				1.00	1.00
Index admission characteristics					
Primary diagnosis					
Yes				0.52 (0.36,0.73)	0.51 (0.36,0.72)
No				1.00	1.00
Current comorbidity (Charlson Index)					
None				1.00	1.00
Mild-moderate				1.42 (0.99,2.02)	1.42 (0.99,2.02)
Severe				2.64 (1.80,3.88)	2.68 (1.82,3.94)
Hospital characteristics					
Hospital peer group					
Principal referral					1.00
Major city					1.32 (0.84,2.08)
District					1.26 (0.76,2.08)
Community					1.11 (0.56,2.22)
Private					0.96 (0.59,1.55)
Others					0.61 (0.24,1.56)
Heart failure service					
Yes					0.90 (0.62,1.29)
No					1.00

*ICC* intraclass correlation coefficient

#### Associations between patient, admission and hospital characteristics and risk of readmission and death

Age-sex adjusted associations between the outcomes and all of the patient, admission and hospital characteristics considered for the multilevel models are shown in Additional file 3: Table S3. The following characteristics were significantly associated with, higher odds of unplanned readmission after adjusting for age and sex: being male; not having private health insurance; lower social interaction scores; severe physical functioning limitations; lower BMI; having previous hospitalisation for: heart failure, other major cardiovascular disease, renal disease or anemia; current comorbidity; increasing hospital admissions and total days in hospital in the previous 12 months; and if the index admission was unplanned or not in a private hospital. A higher odds of 30-day mortality was associated with older age; severe physical functioning limitations; lower BMI; current comorbidity; heart failure as a secondary diagnosis; increasing hospital admissions and total days in hospital in the previous 12 months; and longer length of index admission stay.

In the full multilevel model (Model 5, Table 2), the risk of 30-day unplanned readmission was higher in males (adjusted OR (aOR) = 1.18, 95% CI:1.00–1.37); those with previous hospitalisation for major cardiovascular disease (excl. heart failure) (aOR = 1.44, 1.08–1.91) or for anaemia (aOR = 1.36, 1.14–1.63); and with increasing comorbidity (aOR for severe vs none = 1.26, 1.03–1.54); it decreased with increasing social interaction scores (aOR = 0.95, 0.91–0.99) and increasing BMI (e.g. aOR for obese compared to normal weight = 0.77, 0.63–0.94). Overall, hospital peer group was a significant variable in the model (*p* = .03) with higher odds for public principal, major city, district and community hospitals compared with private hospitals (aOR = 1.33, 1.06–1.68).

In the full multilevel model (Model 5, Table 3), the risk of 30-day mortality was higher in older people, with the odds of post-discharge death increasing 2-fold among those aged 75–84 (aOR = 2.04, 95% CI: 1.24–3.35) and three-fold in those aged 85 or older (aOR = 3.23, 1.93–5.41), compared with those aged 45– < 75 years. It was also higher among those with moderate (aOR = 1.42, 0.99–2.02) or severe comorbidities (aOR = 2.68, 1.82–3.94), compared to none. The risk of risk of 30-day mortality was lower in those with heart failure as the primary rather than secondary diagnosis (aOR = 0.51, 0.36–0.72). The associations between mortality and the two hospital-level variables were not significant.

Characteristics significantly associated with the combined unplanned re-admission/mortality outcome were similar to those for unplanned readmissions models (Additional file 2: Table S2 and Additional file 3: Table S3), reflecting the fact that this combined measure was heavily weighted by readmissions.

Sensitivity analyses showed the direction and size of the associations between patient/hospital characteristics 30-day unplanned readmissions were similar for those with and without prior hospitalisation for heart failure and broadly similar for those with a primary diagnosis only. Due to the small number of events these analyses were not performed for 30-day mortality.

## Discussion

People discharged from hospital with heart failure have relatively poor outcomes. In this study, just over one in ten patients died before discharge, and of those discharged, 4% died within a month and one in five had an unplanned readmission. While unplanned readmission and post-discharge mortality rates did vary across hospitals, in our study this between-hospital variation did not account for a significant proportion of the total variation in outcomes once individual patient characteristics were accounted for. A range of patient characteristics were associated with a higher risk of unplanned readmission, including being male, prior hospitalisation for cardiovascular disease and for anemia, comorbidities at the time of admission, lower BMI and lower social interaction scores. Similarly, risk of 30-day mortality was associated with patient-level factors, in particular age and comorbidity.

Heart failure is one of the most common underlying medical conditions in patients readmitted to hospital within a month of discharge [14, 33]. The risk of unplanned readmission observed in this study was high (21%), and very similar to that reported in a recent NSW report based on initial admissions to all NSW public hospitals (23%) [14]. They are comparable to those reported in a similar study in the UK (18%) [11]. The risks of mortality following heart failure admission and discharge are broadly similar to those reported previously in Australia [4, 5, 7, 17], and internationally [10–12], allowing for differences in methodology, underlying populations and/or changes over time in survival for heart failure. That both sociodemographic factors and clinical factors are independently associated with the outcomes is a consistent finding in previous studies in Australia [4, 5, 7, 17, 18], and elsewhere [10–12, 34, 35].

The considerable patient-level variation in outcomes was not unexpected. More notable from this multilevel study is that, although there was a considerable range in the readmission and mortality risks across hospitals, hospital-level variation accounted for little of the total variation in outcomes. While these findings reflect the variation within our sample and not necessarily the actual variation in outcomes in NSW hospitals across the whole of NSW, they are consistent with those of a recent report on risk-adjusted hospital-level variation in 30-day return to acute care admissions in NSW. Although employing different methodology and only including index admissions to public hospitals, that analysis revealed an overall high readmission rate but few hospital outliers (7% with higher-than-expected readmission rates) after risk-adjustment for patient age, sex and hospital-recorded comorbidities [14]. They are also consistent with the few other analyses that have used multilevel modelling to quantify variation in care, which found hospital-factors explained very little of the variation in 30-day readmissions in the US [13] and 30-day mortality in Sweden [12].

Taken together, the study findings indicate that the issue of high readmission rates is a system-wide one, rather than an issue confined to particular hospitals. Moreover, considering the very high levels of morbidity and frailty associated with heart failure, it is likely that many of the factors influencing the risk of readmission reside beyond the sphere of the hospital. Indeed, it could be the case that the high readmission rates in heart failure patients reflect a more general phenomenon, that of frequent admissions among elderly people with multiple chronic conditions. Certainly, levels of comorbidity and physical functionaling impairment were substantial among heart failure patients in this study and were strongly associated with readmission. That comorbidity is a key predictor of readmission is consistent with previous studies of heart failure patients [5, 7, 11, 14], and other patient populations, such as those who have had a stroke [36].

The study findings reinforce the importance of considering patient-level factors when interpreting standard performance measures, such as short-term readmissions. A recent US study has shown that if these factors are not adequately accounted for, it can lead to perverse outcomes, where higher quality hospitals that serve vulnerable or medically complex patient populations may be unfairly penalised for apparently poor performance [37]. Moreover, readmission rates are only likely to be a reasonable indicator of quality of care if there is considerable variation in the rates and if unmeasured case-mix variations account for a small proportion of the inter-hospital differences in these rates [38].

This is not to say that health systems are powerless to decrease readmission rates. A recent Cochrane review of 25 randomised controlled trials including 5942 patients found case management interventions, including nurse monitoring of patients post-discharge by phone and home visits, and multidisciplinary interventions that promoted coordinated care of heart failure patients after discharge, were associated with a reduction in both all-cause and heart-failure-related readmissions [39]. Included in these reviews were two Australian trials, which demonstrated a reduction in unplanned readmissions and deaths in those receiving follow-up at home from a cardiac nurse with multidisciplinary input [40, 41]. Further Cochrane reviews concluded that telemonitoring (remote monitoring of vital signs by cardiac specialist) and structured telephone support interventions reduce mortality and heart failure-related hospitalisations when compared to usual care [42], and that exercise based-rehabilitation programs for heart failure reduce the risk of heart failure-specific hospitalisation [43]. Certain features of heart failure disease management programs, such as in-person communication, intensive patient education and self-care supportive strategy, medication optimization, and active involvement of a cardiac nurse and cardiologist, have been identified as components in successful programs that reduced hospital readmissions and deaths related to heart failure and improved health outcomes [44, 45]. Further, although prospective studies are lacking, extensive expert opinion and consensus has been published regarding the importance of multidisciplinary palliative care [46].

In this study, by linking hospital and death data to baseline survey data we were able to model variation at both the hospital and patient level, taking into account a large range of patient sociodemographic and health characteristics. However, there are several limitations that should be borne in mind when interpreting the results: (i) While participants were randomly sampled, hospitals were not; (ii) Data on exposures were mostly based on self-report; (iii) Several of the health measures were baseline rather than contemporaneous with the admission, and the elapsed-time between baseline health status and the index admission varied across patients; (iv) There was a lack of measured clinical data (e.g. blood test and echocardiogram results); (v) There was insufficient power to separately analyse index admissions according to whether the diagnosis was primary or secondary, readmissions exclusively for heart failure, and shorter-term outcomes. It should also be noted that despite the large overall sample size, power was limited for some of the specific comparisons so negative null findings should be interpreted with caution; (vi) While we had information on the presence of a dedicated heart failure service supplied by the NSW Heart Foundation, the completeness of this information has not been verified; and (vii) While the 45 and Up cohort are broadly representative of the Australian population in this age group, the cohort does not include people in residential care facilities, and study participants are likely to be healthier and have lower absolute hospitalisation rates than the general population in this age group. However, given the near-complete follow up of the cohort and other methodological considerations, internal associations between patient, admission and hospital characteristics are considered valid [47, 48].

A general, but important, limitation to consider when using hospital data to report on heart failure is the accuracy of the hospital coding for heart failure diagnoses. A recent validation study using NSW APDC data showed relatively high positive predictive values, of between 85 and 92% [49], meaning that while we may not have picked up every patient in the cohort who was admitted with heart failure in the follow-up period, those who we did include are likely to have had heart failure. Finally, of particular interest to clinicians and policy makers is whether the type of clinician providing care (e.g. general specialist vs cardiologist) is associated with patient outcomes, but this information is not available in the hospital data. It would be also useful to have more detailed information on hospital characteristics and post-discharge out-of-hospital care.

## Conclusions

Findings suggest that the issue of high rates of readmission and mortality in people with heart failure apply more to the system as a whole than to particular hospitals. That comorbidity is a major predictor of both readmission and mortality highlights the need for caution in penalising hospitals for treating complex patients. They also point to the need for patient-centred strategies to identify those at high risk of readmission and to optimise heart failure management, including appropriate discharge planning and accessible community services. The findings also reinforce the importance of enhanced preventative strategies as the population ages, risk factor burden increases and survival rates from acute coronary syndromes continue to improve.

## Additional files

Additional file 1: Table S1. Hospital characteristics. (XLSX 10 kb)
Additional file 2: Table S2. Multilevel model results for combined 30-day unplanned readmission/mortality: random effect measures and adjusted odds ratios (95% CIs) for individual-level and hospital-level variables. (XLSX 15 kb)
Additional file 3: Table S3. Age-sex adjusted associations between patient, index admission and hospital characteristics and each of the outcomes. (XLSX 15 kb)

## Abbreviations

aOR: Adjusted odds ratio
BMI: Body mass index
CI: Confidence interval
ICC: Intraclass correlation coefficient
ICD-10: International Classification of Diseases 10th revision
IQR: Interquartile range
K10: Kessler Psychological Distress Scale
NSW: New South Wales
OR: Odds ratio
RBDM: Register of Births, Deaths and Marriages
